# Ocular complications of oak processionary caterpillar setae in the Netherlands; case series, literature overview, national survey and treatment advice

**DOI:** 10.1111/aos.14607

**Published:** 2020-09-30

**Authors:** Matthew K. H. Tan, Maarten B. Jalink, Naïlah F. M. Sint Jago, Lintje Ho, J. H. Arnold van Vliet, Tridib Das, Jan Tjeerd H. N. de Faber, Robert P. L. Wisse

**Affiliations:** ^1^ Department of Ophthalmology University Medical Center Utrecht Utrecht The Netherlands; ^2^ Dutch Expertise Center for the Oak Processionary Caterpillar Wageningen University and Research Wageningen The Netherlands; ^3^ Rotterdam Eye Hospital Rotterdam The Netherlands

**Keywords:** caterpillar hair, exotic species, oak processionary caterpillar, setae, thaumetopoea

## Abstract

During early summer 2019, the Netherlands experienced an outbreak of the exotic oak processionary caterpillar. The vast number of caterpillars, which live in large nests on oak trees before they turn into moths, possess thousands of small, barbed hairs (setae) that are disseminated with the wind. The hairs cause a range of primarily dermatologic problems. However, Dutch ophthalmologists started reporting patients with ophthalmologic complaints caused by the penetrating hairs of the oak processionary caterpillar. This paper focuses on the ophthalmologic complications caused by the caterpillar hairs. We collected a series of four cases with reports ranging from a corneal erosion with hairs lodged into the cornea, to a sterile endophthalmitis in which hairs were found in the vitreous. A literature review for similar cases was performed using the PubMed and Embase database. Together with the Dutch Ophthalmic Society (Nederlands Oogheelkundig Gezelschap, NOG), a national survey was issued to determine the scale of this new problem. This showed that oak processionary caterpillar related complaints are primarily limited to the south of the Netherlands. Suggested ophthalmic treatment guidelines are presented. With the next summer at the doorstep, and limited preventative measures against the caterpillar hairs, we expect a new wave of ophthalmologic complaints coming year as well.

*Thaumetopoea processionea* (order *Lepidoptera*; family *Thaumetopoeidae*), colloquially known as the oak processionary moth, is a type of butterfly that occurs naturally in southern Europe, but has recently expanded in range with populations in Northern and Western Europe. Climate change has been linked with its expansion, albeit through unknown mechanisms. Countries whose climate is influenced by the Atlantic Ocean, such as Belgium, the Netherlands and Germany, more frequently report outbreaks of the larvae of the *T. processionea*, the oak processionary caterpillar (Maier et al. 2003). As recent as the summer of 2019, the Netherlands experienced its worst outbreak in years as reflected by the number of people presenting at their general practitioners with health‐related complaints resulting from the outbreak, at one point peaking at 110 per 100 000 inhabitants over the period of 1 week (Hooiveld et al. 2019).

**Figure 1:**
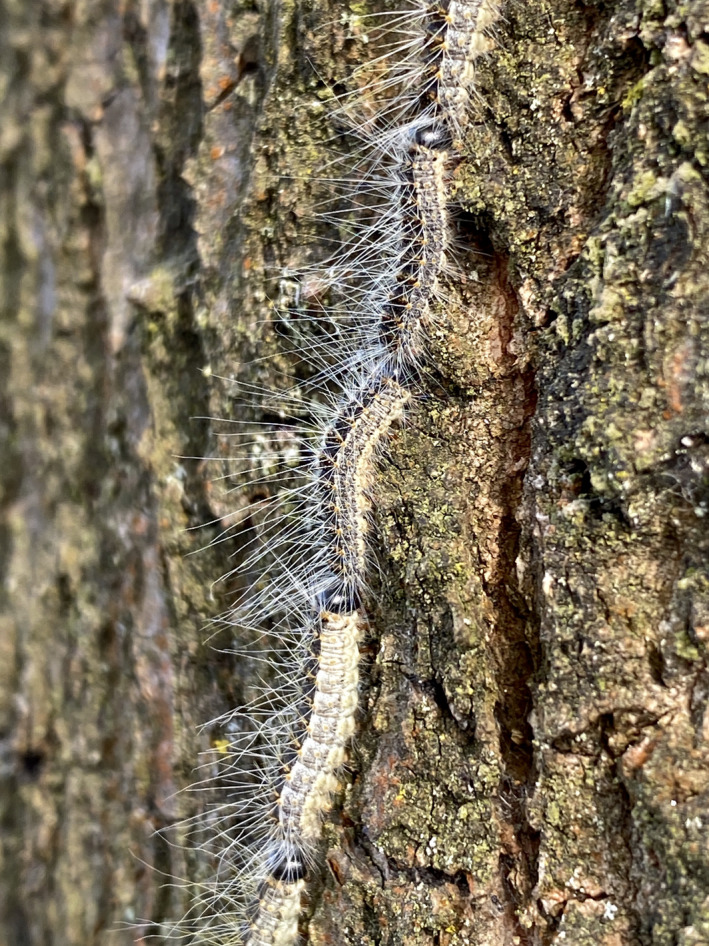
The oak processionary caterpillar. Note the many smaller hair attached to the body.

These outbreaks have a significant impact on human health, mainly due to the airborne spread of thousands of small (0.1–0.2 mm) urticating hairs, known as setae (Fig. 1). The setae are released by the larvae from the third instar onward (Gottschling & Meyer 2006), which begins around the month of May. The setae contain thaumetopoein, a histamine releasing toxin which can retain their allergenic activity even several years after an outbreak (Maier et al. 2003). Lepidopterism occurs upon exposure of skin or mucosa to these hairs (Hossler 2019). Depending on the exposed region, complaints can manifest as a variety of systemic or local reactions such as respiratory difficulty, ocular problems, sore throats and skin irritation (Rahlenbeck & Utikal 2015).

With much of the recently reported medical literature describing dermatologic complaints, this paper presents a novel set of cases of ophthalmological significance. In collaboration with the Dutch Expertise Center for Oak Processionary Caterpillars chaired by Wageningen University, together with the National Institute for Public Health and the Environment (Rijksinstituut voor Volksgezondheid en Milieu, RIVM), the authors aim to inform professionals and the general public alike on ophthalmological insights of the oak processionary caterpillar (Fig. 2).

**Figure 2:**
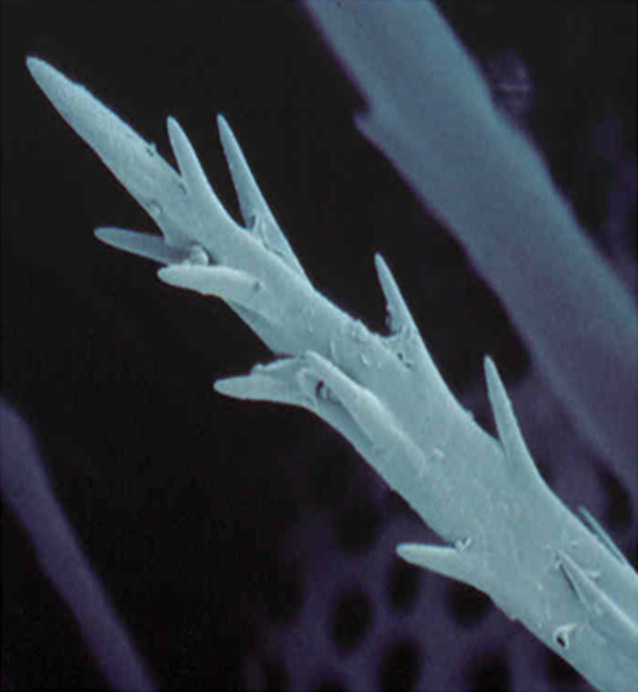
Scanning electron microscope image of an oak processionary caterpillar seta. (Photograph by Henk Jans).

This paper consists of four components: a case series, a literature overview, the results of a national survey among ophthalmologists and a treatment advice.

## Cases series

*Patient A* is a 59‐year‐old, healthy man from the south of the Netherlands. He was struck in the right eye with an unknown object while cycling along an oak forest, known to be infested with oak processionary caterpillars. At the emergency department, only a small erosion was noticed and a bandage with antibiotics was applied. Due to persisting pain a day later, the patient was referred to a general ophthalmologist.

**Figure 3:**
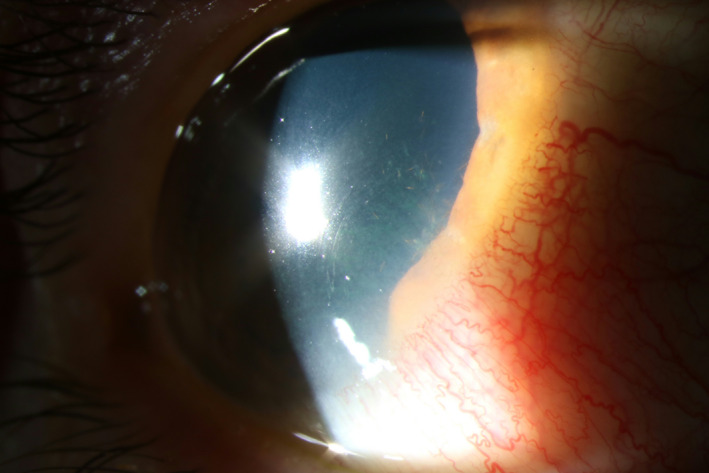
Archetypical oak processionary setae‐associated keratoconjunctivitis with localized redness with setae visible in the corneal epithelium. Mild fluorescein staining can be seen, with a quiet anterior chamber.

The main complaints were itching and photophobia in the right eye. Visual acuity was normal. On slit‐lamp examination, more than 30 small hairs were seen in the cornea on the inferonasal side (Fig. 3). The processionary setae were already lodged in the corneal stroma and could not easily be removed. Therefore, patient was prophylactically treated for possible pathogens on the hairs, including fungi; topical ofloxacin and chlorhexidine were given four times a day.

Two weeks later, the patient was free of complaints. The setae had remained in their exact same position. There was no aqueous reaction, nor had limbal nodules developed. Topical medication was stopped hereafter.

*Patient B* is a 46‐year‐old man with complaints of red and itchy eyes. Interestingly, he had red, itching bumps all over his body as well. This happened after he walked through the forest a day prior to presentation at the outpatient clinic. Our patient had normal visual acuity. Nasal hyperaemia in the left eye was the only pathological finding that could be assessed on slit‐lamp examination, for which he was treated with topical ofloxacin and chlorhexidine, both six times a day. After a week, there were no complaints and the drops were discontinued.

*Patient C* is a 56‐year‐old man that presented with a red right eye with good visual acuity and multiple red spots on his arms. The day before presentation, he biked through a forest known to be invaded by oak processionary caterpillars. Slit‐lamp examination showed hyperaemia and chemosis of the conjunctiva with several setae lodged in the cornea. The setae in the epithelium were removed by a hockey‐stick knife. As before, topical ofloxacin and chlorhexidine, both six times a day, were given. In 3 days, the clinical state had improved and after 2 weeks there were no complaints and the drops could be discontinued.

*Patient D* is a 70‐year‐old man that was referred with progressive decline of visual acuity in his left eye since 2 months. He was treated with anti‐VEGF because of exudative age‐related macular degeneration. Six weeks after injection, he noted a decline in visual acuity, which was 0.05 (Snellen) at presentation. On slit‐lamp examination, hyperaemia of the conjunctiva, 2+ cells in the anterior segment, no flare and 4+ cell in the vitreous were found. Also, three thin, hair‐like structures were seen floating in the vitreous. Fundoscopy only showed a red reflex.

**Figure 4:**
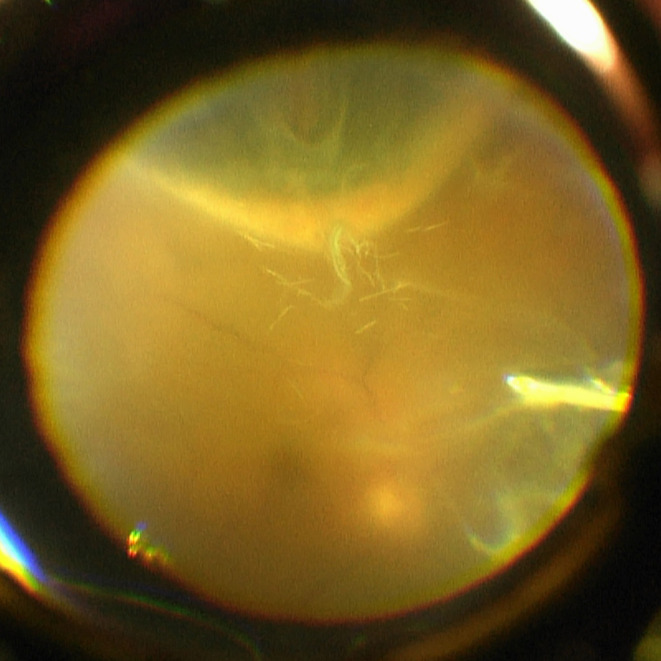
Several hair‐like structures lodged in the vitreous can be clearly seen on indentation, as viewed during pars plana vitrectomy. (Videostill by Lintje Ho).

During subsequent pars plana vitrectomy, around ten hairs were seen in the vitreous, with haze and lots of inflammatory cells (Fig. 4). Intravitreal antibiotics and subconjunctival steroids were given. Additional anterior chamber tap and culture of the vitreous showed no pathogens.

The vitreous was sent for pathological examination, and oak processionary setae were collected from a nest in an oak tree near the hospital by a brave pathology resident for comparison. Typical processionary setae could not be identified in the vitreous, due to the lack of the characteristic barbs (Maier et al. 2003). This could be the result of the hairs being in vitreous inflammation for 2 months (Steele et al. 1984; Fraser et al. 1994).

With all other diagnoses excluded and the ongoing outbreak of oak processionary caterpillars, this case of endophthalmitis was concluded to be caused by a reactive uveitis to processionary setae (RUPS). On follow‐up, visual acuity remained low (0.1), possibly because of other ocular morbidity.

## Literature overview

A search in the PubMed and Embase databases was performed, using the terms ‘oak processionary caterpillar’, ‘oak processionary moth’, and ‘*Thaumetopoea processionea*’. All papers were screened on title and abstract for ophthalmologic papers. In total, 36 (PubMed) and 42 (Embase) paper were found and retrieved.

There is a limited number of published articles regarding the subject; much of what has been published is in the realm of dermatology and allergology. No literature with ophthalmological relevance was found.

The pine processionary moth (*T. pityocampa*), a relative from the same genus, however, was featured in several case reports over the last 10 years. Portero et al. described cases of comparable nature with patients A and C, where the resulting injury was confined to the cornea (Portero et al. 2013). Case reports by Rajagopalan et al. (2020) and Blasetti et al. (2012) provide evidence that caterpillar setae are capable of penetrating the cornea and affecting visual acuity, thus supporting the diagnosis of patient D even though pathological examination was unverifiable in that instance.

## National survey

During the outbreak in July 2019, the Dutch Ophthalmic Society (Nederlands Oogheelkundig Gezelschap, NOG), sent out an online survey to all ophthalmologists in the Netherlands to determine the severity of the outbreak. All responses that were received within one week were included in the data analysis, which resulted in total 156 ophthalmologists responding with 196 involved patients. Response to the survey was voluntary and could be completed in less than ten minutes. The survey consisted of eight questions and encompassed qualitative and quantitative questions to determine whether ophthalmologic cases due to the oak processionary caterpillar were seen, and if so, which presenting symptoms, part of the eye, and treatment were involved. To determine the geographic location of each response, an IP address of the responder was documented. No statistical tests were performed.

The distribution of cases was largely received from the two southern Dutch provinces (North Brabant and Limburg), based on IP address of the respondents. There were no cases reported in the three most northern provinces of Drenthe, Friesland and Groningen.

Among the respondents, almost half (49%) reported a hyperaemic conjunctiva as the initial symptom, followed by pain and itchiness in 33% of respondents, whereas vision loss was reported in ~9%. Topical corticosteroids and topical antibiotics were the preferred medical treatment, as both were administered by one third (~30% and ~35%, respectively) of respondents. In total, 5% of respondents reported of having seen patients with lasting damage, including vision loss or stray light complaints.

## Treatment advice

Based on clinical experiences, the Dutch Cornea Society issued a first treatment advice for patients that consult the general practitioner (primary care) and ophthalmologist (secondary care) with presumed sequelae of *T. processionea*.

Guidelines for treatment in primary care:

Avoid areas endemic with oak processionary caterpillars. Rinse briefly with water, do not rub. Treat purulent secretion with chloramphenicol eye ointment three times daily for 1 week. In case of loss of vision, complaints >48 h and severe photophobia refer to an ophthalmologist.

Guidelines for treatment in secondary care:

Primarily remove as much hairs/setae as possible, consider local abrasion of corneal epithelium. Hairs can penetrate deep into stroma and even anterior eye chamber; these are practically impossible to remove.

- In case of severe inflammatory symptoms (chemosis, photophobia, limbitis): tavegil 1mg once, then levocetrizine 1dd5mg for 2 weeks and topical steroids (fluormetholone/hydrocortisone).
- In case of signs of complicated infection (corneal infiltrates, purulent discharge, anterior uveitis): ofloxacine 8dd for 2 weeks and short follow‐up. Consider chlorhexidine 0.02%.
- The hairs may remain in the stroma for a very long time (up to 12 months). In case of persistent inflammation: treatment with mild topical steroids (fluormetholone/hydrocortisone).

The oak processionary moth poses a novel public health risk with frequent and potentially severe ophthalmic side effects. Its setae (hairs) often attach to the conjunctiva and corneal epithelium and penetrate into the eye in severe cases. A comprehensive strategy to combat the oak processionary caterpillar is not yet currently available, and avoiding oaks is virtually impossible in daily practice.

Clinically, most ophthalmologic cases of oak processionary caterpillar complications are self‐limiting, but severe cases with long‐lasting sequelae are reported. This manuscript details ophthalmic manifestations, based on expert opinion after one severe season. An extensive survey on the epidemiology and clinical sequelae of the oak processionary caterpillar is anticipated in the summer of 2020.
